# Supplementary material for: Quantile granger causality between clean energy and tourism stock indices: Evidence from regional markets
Source: PLoS One. 2025 Jun 23;20(6):e0326256. doi: 10.1371/journal.pone.0326256 (PMC12184921; doi:10.1371/journal.pone.0326256)
Supplement: Attachment — Submitted filename: Review_to_Authors.docx [file pone.0326256.s002.docx]

**QUANTILE GRANGER CAUSALITY BETWEEN CLEAN ENERGY AND STOCK MARKET TOURISM INDICES: REGIONAL ECONOMETRIC EVIDENCE**

**FIRST READ-THROUGH**

**Main goals:** to analyze the relationship between clean energy and tourism stock market indices in major global economies. More specifically, to: (i) determine whether a long-run and short-run relationship exists between the WilderHill Clean Energy Index and tourism stock indices from the United States, Europe, China, and Australia; (ii) assess whether the direction and nature of causality between these sectors vary across regions and market conditions; (iii) understand the regional factors that influence these relationships, such as policy frameworks or economic structures; and (iv) analyze how global economic shocks, particularly COVID-19, have altered these intersectoral dynamics.

**Methods:** (i) Stationarity tests: Augmented Dickey-Fuller (ADF), Phillips-Perron (PP), KPSS, Fourier Unit Root, and quantile Augmented Dickey-Fuller (QADF) to assess stationarity at different quantiles and in the presence of structural breaks; (ii) Causality tests, precisely the traditional Granger causality to establish baseline linear relationships, and the quantile Granger causality to capture nonlinear and conditional dependencies across various quantiles (i.e., market states); (iii) Time-frequency analysis, precisely the wavelet coherence analysis to evaluate time-frequency interactions between indices, capturing dynamic and periodic comovements.

These methods allow for addressing the data's static and dynamic, linear and nonlinear characteristics.

**Data:** the data spans from January 1, 2010, to December 31, 2023, and includes five variables: one clean energy index [WilderHill Clean Energy Index (LWILDER)], and four tourism-related indices [STOXX Europe 600 Travel & Leisure Index (LSTOXXR); Dow Jones U.S. Travel & Leisure Index (LDJUSTT); FTSE China A 600 Travel & Leisure Index (LFTXIN); S&P/ASX 300 Hotels Restaurants & Leisure Index (LAXHRKD)].

The frequency of the data and the number of observations considered should be identified.

**Main results:** (i) multiple stationarity tests confirmed that all time series are I(1); using traditional Granger causality test, no significant causality was found; (iii) the quantile Granger causality revealed: significant causality from clean energy to U.S., European, and Australian tourism indices at extreme quantiles (especially during high volatility), and that the direction and intensity of causality are region-specific and conditional on market state; (iv) the wavelet coherence analysis revealed: stronger medium- and long-term comovements between clean energy and European/Australian tourism stocks, weaker and more transient links with the U.S. index, consistent with economic independence and post-pandemic resilience, and that COVID-19 produced synchronized, high-frequency movements across regions.

This manuscript falls within PLOS ONE’s interdisciplinary and methodologically rigorous work scope.

The paper includes recent and relevant references, but only about 49% are from the last five years. Thus, the literature review should be updated with more recent studies.

The experimental design is well-suited to achieving the study’s objectives.

The figures and tables are appropriate. However, figures (wavelet plots) could benefit from more explicit legends explaining phase arrows and highlighting significant zones with clearer annotations.

The conclusions are consistent with the reported results. However, while the conclusions are relevant and potentially useful, they sometimes overstate the generality of the findings. Traditional causality tests were not significant, and causal patterns appeared only in specific quantiles or periods. A more balanced tone would better reflect the conditional nature of the findings. Furthermore, it should be helpful to add a brief section on policy and investment implications (e.g., how these results might guide investment strategies in regions where tourism is more sensitive to clean energy trends).

Generally, the English language is appropriate and understandable. However, there are minor grammatical issues, and it is recommended to have a professional English editing for clarity, conciseness, and consistency (especially in the methods and discussion sections).

**DETAILED REVIEW**

**TITLE**

The title reflects the core econometric technique (quantile Granger causality) and the variables studied (clean energy and tourism stock indices). However, “regional econometric evidence” is generic. Suggestion: *Quantile Granger Causality Between Clean Energy and Tourism Stock Indices: Evidence from Regional Markets.*

**KEYWORDS**

The current keywords are technically relevant but imprecisely formatted and overly narrow (e.g., “Fourier-Financial markets sustainability-Wavelet” appears as one compound term). Suggestion: *Clean Energy, Tourism Stock Indices, Quantile Granger Causality, Wavelet Coherence, Sustainable Finance, Regional Econometrics.*

**ABSTRACT**

The abstract partially summarizes the study's goals, methods, and key findings. The goals and general approach are described, but the abstract should also include information about the study period analyzed, as well as the frequency of the data. It should also briefly explain the implications of the findings for tourism and clean energy investment.

Furthermore, to maximize the article’s impact, it should highlight the contributions and implications of the study. The abstract's first sentence should be about the problem, motivation, or question. The second sentence should be about what we already know from the literature. In the third sentence, please say what you are doing and how; the following two sentences should be about what you find, followed by the significance of your findings. It is important to say what the question is; why this is an interesting and important question; how you go about it; why this is a persuasive modeling strategy; what you found; how this changes our view of the world (focusing on its novelty in relation to what we already know from the literature). People only spend one or two minutes reading an abstract; if they do not see the novelty within that time, they tend to ignore a paper. Our aim is to ensure that your paper gets cited after publication. Please consider highlighting the big ideas clearly and succinctly that is informative, novel, logical, and engaging. A reader will not be interested only in what you found but why you found it and how it helps solve a problem with more focus on practical implications/economic story rather than pure technical results; if you can add why you find something or why you have done something, they can have a bigger impact than just saying what the paper is doing.

**INTRODUCTION**

This section defines the thematic intersection of clean energy and tourism, positioning it within the context of global sustainability efforts. It also raises policy-relevant questions, referencing COP-26, ESG investment, and post-COVID recovery. However, this section requires improvements considering the following:

1. The core research questions are only explicitly listed later (Section 2.4), making the narrative arc in the introduction less focused than it could be. Please, try to move the explicit research questions (currently in Section 2.4) into the final paragraph of the introduction.
2. The introduction contains redundant generalities, such as repeated mentions of tourism's importance to GDP, without quantifying or differentiating regionally. Streamline paragraphs to reduce general statements and focus more on the literature gap motivating this study.
3. No hypotheses are clearly stated, even though the rest of the paper explores directional and quantile-based relationships. Try to formulate explicit hypotheses (e.g., *We hypothesize that clean energy indices Granger-cause tourism indices in developed economies under extreme market conditions*.)

**BACKGROUND LITERATURE**

This section attempts to contextualize the relationship between energy, sustainability, and tourism markets. It cites a broad range of sources, some relatively recent (e.g., 2024, 2025). However, only about 49% of the references in the manuscript are from the last five years. Thus, this section should be updated with more recent references. Furthermore, the literature review is broad but not analytical. It lacks critique, comparisons, or synthesis of findings across studies. Finally, the contribution of this study relative to the literature is not clearly articulated. Please, make a more explicit case for originality, highlighting what is *not yet known* and how this study addresses it.

**DATA AND MODEL SPECIFICATIONS**

The data sources are stated, and transformations (log differences, etc.) are appropriately described. A range of econometric tools is introduced, showcasing methodological sophistication. However, this section also requires improvements, considering the following.

1. The frequency and source details about the tourism-related indices are missing. Please, improve it.
2. There is no justification for the specific 2010–2023 time frame (why 2010 was chosen as a starting point? Why is the end date December 31, 2023?).
3. Justify the choice of indices and the countries/regions selected. Why not Japan, or emerging markets?
4. Add a table of descriptive statistics (means, standard deviations, skewness, kurtosis) to offer basic intuition about the series.
5. Explain why wavelet coherence and quantile causality are superior to traditional methods for this research context.

**EMPIRICAL RESULTS**

This section provides a detailed account of causality relationships across quantiles and time frequencies. The use of wavelet coherence plots adds valuable visual information about dynamic interdependence. However, this section also requires improvements, considering the following.

1. Results are reported without sufficient interpretation, i.e., the text often restates what the tables show. Please add a brief narrative interpretation of each result table. What does causality at the 0.05 quantile mean in practical terms?
2. Wavelet coherence figures lack clear legends and annotations. Please improve wavelet figure readability by adding explanations of arrows (phase differences) and clarifying high-power zones.
3. Phases are not explained for non-expert readers.
4. Check if this information is correct: “*%0.10 ***%0.01 **%5”
5. For clarity, consider plotting a summary heatmap of significant quantile causality results across regions.

**DISCUSSION AND ECONOMETRIC FINDINGS**

The discussion begins to link findings to regional policy contexts (e.g., Australia’s ESG investment climate). It acknowledges the time-varying nature of relationships and the impact of COVID-19. This section also requires improvements, considering the following.

1. The discussion largely repeats results without much deeper theoretical interpretation. Interpret results through a theoretical lens, such as the environmental Kuznets curve or portfolio diversification theory.
2. There is no reference to economic mechanisms (e.g., why might clean energy investment lead tourism stock performance in the U.S. but not in China?).
3. It lacks a comparative discussion with existing literature. Do these results confirm or contradict previous studies? Compare findings with other empirical studies on energy–tourism links to contextualize significance.

**CONCLUSION AND RECOMMENDATIONS**

The conclusion restates the broad main goal of analyzing the interrelationship between clean energy and tourism stock indices using time-frequency and quantile-based approaches. It emphasizes the importance of these findings for policymakers and investors, particularly in the context of COVID-19 recovery and ESG priorities. It also reiterates that regional differences were found in causality patterns, consistent with the evidence presented. However, this section should also be improved considering the following.

1. The “recommendations” section remains broad and general, lacking direct, actionable suggestions for different stakeholders. Make recommendations actionable, specifying who should act and how.
2. There is no differentiation between what should be done by governments, tourism sector actors, or investors. For instance, how might asset managers in clean energy funds use this information?
3. Acknowledge study limitations transparently, e.g., the analysis is limited to four regional markets, excluding potentially relevant emerging economies such as Brazil or India.
