# Supplementary material for: Quantile granger causality between clean energy and tourism stock indices: Evidence from regional markets
Source: PLoS One. 2025 Jun 23;20(6):e0326256. doi: 10.1371/journal.pone.0326256 (PMC12184921; doi:10.1371/journal.pone.0326256)
Supplement: Attachment — Submitted filename: Response to Reviewers2.docx [file pone.0326256.s004.docx]

**Response to Reviewer 1**

We sincerely thank Reviewer 1 for their detailed and constructive feedback. Below, we provide a point-by-point response to each comment. Where appropriate, revisions have been made throughout the manuscript to reflect the suggested improvements.

1. **Identification:** The fundamental flaw lies in the inability to establish proper identification for causal relationships between clean energy and tourism indices. While the term "causality" is used extensively throughout the paper, what is presented is merely statistical predictability rather than true causal effects. The Granger and quantile Granger causality approaches employed are insufficient for establishing causal relationships in financial markets characterized by simultaneous determination of prices and complex feedback mechanisms. The paper fails to address the critical issue of omitted variables bias. Both clean energy and tourism stock indices are likely influenced by common macroeconomic factors (e.g., interest rates, economic growth, inflation), market-wide sentiment, and global shocks. Without controlling for these common drivers, the apparent "causal" relationships may simply reflect spurious correlations driven by omitted variables rather than true structural relationships between these sectors. A rigorous identification strategy would require instrumental variable approaches, natural experiments, or structural models that can account for simultaneous determination. For example, the paper could exploit exogenous policy changes (like renewable energy subsidies) that directly affect clean energy stocks but affect tourism stocks only through their impact on clean energy markets. Such approaches are essential for journals of this caliber. To improve the causal identification, the paper should consider methodologies similar to those in Rigobon (2003) and Nakamura and Steinsson (2018). Rigobon's identification through heteroskedasticity could help separate the bidirectional effects between clean energy and tourism indices. Additionally, incorporating structural VAR models with appropriate restrictions based on economic theory would strengthen the identification strategy and improve causal interpretations.

We sincerely thank Reviewer 1 for the insightful comments regarding the identification of causal relationships and the limitations of Granger-based methods in this context. In response, we have expanded our empirical analysis by incorporating a Structural VAR model, which provides a more theoretically grounded framework to examine the dynamic linkages between clean energy and tourism stock indices. While this addition improves the interpretability and robustness of our findings, we fully acknowledge that certain endogeneity concerns such as omitted variable bias or simultaneity may still remain. To reflect this, the revised manuscript explicitly discusses these methodological limitations and emphasizes the need for future research to adopt more rigorous identification strategies, including heteroskedasticity-based approaches (Rigobon, 2003), instrumental variables, or natural experiments exploiting exogenous policy shocks. We believe these clarifications have significantly enhanced the conceptual precision and transparency of the study. (Model specifications (p. 17, L. 596), Empirical Results (pp. 26 – 30).

2. **Methodology:** The paper employs an extensive battery of econometric techniques without sufficient justification for their selection or consideration of their limitations. There are several serious methodological issues: First, the implementation of the quantile Granger causality test is problematic. The paper reports results from quantile regressions but does not adequately address the inference issues associated with these methods in time-series contexts. Unlike OLS-based Granger causality, quantile-based inference requires appropriate bootstrap procedures to account for dependence structures in the data. The paper does not discuss how standard errors are computed for the quantile causality tests, raising concerns about the validity of the statistical significance reported. Second, the wavelet coherence analysis presents results without addressing critical limitations. The interpretation of wavelet coherence diagrams is largely subjective, yet the paper makes strong claims about lead-lag relationships based on phase differences without formal statistical testing. As noted by Aguiar-Conraria and Soares (2014), wavelet coherence can produce misleading results when common factors drive both series. The paper fails to implement significance testing for wavelet coherence or control for potential confounding factors. Third, the unit root testing procedure appears inconsistent. Table 5 shows that most series are non-stationary at levels across quantiles, yet the subsequent analysis sometimes treats these series as if they were stationary. The implications of non-stationarity for the quantile causality analysis are not adequately addressed, potentially leading to spurious relationships being identified as causal.

We thank the reviewer for raising this important methodological issue. In the revised manuscript, we explicitly acknowledge that the quantile Granger causality test is estimated using Huber sandwich standard errors and kernel-based sparsity estimation (p. 26, L. 725). As noted in the revised empirical section, the model does not implement bootstrap-based inference. Therefore, we have added a methodological caveat noting that the reported p-values may not fully capture distributional uncertainty under serial dependence or heteroskedasticity. We agree that future applications of quantile causality tests should incorporate bootstrap methods to ensure more robust inference, and we have clearly indicated this limitation and recommendation in the updated text.
We also appreciate the reviewer’s observation regarding the interpretive limitations of the wavelet coherence analysis. In the revised manuscript (p. 25, paragraph 3), we now clearly state that although high-coherence regions are highlighted, they do not provide formal statistical evidence of causality or directionality due to the absence of simulation-based significance testing. Following the methodological caveats emphasized by Aguiar-Conraria and Soares (2014), we interpret the wavelet results with caution.

At the same time, we justify the inclusion of wavelet coherence analysis as an exploratory technique that offers unique insights into time–frequency dependencies and evolving lead–lag structures that are often overlooked in conventional time-domain analyses. This addition helps to visualize the dynamic and asymmetric nature of the relationship between clean energy and tourism stock indices, thereby enriching the overall analytical contribution of the study.

Finally, we thank the reviewer for highlighting the crucial role of stationarity in time-series causality analysis. In response, the revised manuscript includes extensive unit root testing using traditional (ADF, PP, KPSS), Fourier, and quantile-based (QADF) procedures. The results confirm that all series are integrated of order one, I(1). Accordingly, all causality analyses—including quantile causality—are conducted on the first-differenced series, as clearly stated in the empirical methodology and result tables. This ensures consistency with the stationarity requirements and improves the robustness of our inferences (p. 21–22).

**3. Theory:** The paper lacks a solid theoretical framework to justify the expected relationships between clean energy markets and tourism stock indices. While the literature review mentions various studies on energy, tourism, and financial markets, it fails to develop a coherent economic model explaining why and how these specific sectors should be interconnected. The literature gap identified on page 7 acknowledges that "few studies have considered the effects of clean energy investments and clean energy indices on tourism stock indices," but does not explain why this relationship is economically meaningful or important to study. Without a clear theoretical mechanism linking these sectors (beyond vague references to sustainability), it is difficult to interpret the empirical findings or understand their implications. Furthermore, the economic interpretation of the empirical results is superficial. For instance, the quantile causality results in Table 10 show various significant relationships across different quantiles, but the paper provides little economic rationale for why causality would exist at some quantiles but not others. Similarly, the wavelet analysis identifies time-varying relationships without connecting these patterns to specific economic events or structural changes in the markets beyond general references to the COVID-19 pandemic. The paper should develop a theoretical model in line with approaches used by Pástor et al. (2022), who provide a theoretical framework for understanding the pricing and impact of ESG factors in financial markets. Such a model would help contextualize why clean energy indices might predict or be predicted by tourism indices, and would provide a foundation for interpreting the empirical findings beyond mere statistical associations. References Aguiar-Conraria, L., & Soares, M. J. (2014). The continuous wavelet transform: moving beyond uni- and bivariate analysis. Journal of Economic Surveys, 28(2), 344-375. https://imsva91-ctp.trendmicro.com:443/wis/clicktime/v1/query?url=https%3a%2f%2fdoi.org%2f10.1111%2fjoes.12012&umid=E820DE85-345F-6D06-B64E-20391C1672CC&auth=3c436cd85e6f7f7a73ca8613b640f4e6e0edaf42-a240a3bda9ef1ef3e297408ad6554b58e736b24c Pástor, L., & Stambaugh, R.F. & Taylor, L.A., 2021. Sustainable investing in equilibrium. Journal of Financial Economics, 142(2), 550-571. https://imsva91-ctp.trendmicro.com:443/wis/clicktime/v1/query?url=https%3a%2f%2fdoi.org%2f10.1016%2fj.jfineco.2020.12.011&umid=E820DE85-345F-6D06-B64E-20391C1672CC&auth=3c436cd85e6f7f7a73ca8613b640f4e6e0edaf42-8517b137b8b3fa218520adaf018713ecbfba9640 Nakamura, E., & Steinsson, J. (2018). Identification in macroeconomics. Journal of Economic Perspectives, 32(3), 59-86. https://imsva91-ctp.trendmicro.com:443/wis/clicktime/v1/query?url=https%3a%2f%2fdoi.org%2f10.1257%2fjep.32.3.59&umid=E820DE85-345F-6D06-B64E-20391C1672CC&auth=3c436cd85e6f7f7a73ca8613b640f4e6e0edaf42-2c5f98c8547afec6c573143351d6645f47becde6 Rigobon, R. (2003). Identification through heteroskedasticity. Review of Economics and Statistics, 85(4), 777-792. <https://imsva91-ctp.trendmicro.com:443/wis/clicktime/v1/query?url=https%3a%2f%2fdoi.org%2f10.1162%2f003465303772815727&umid=E820DE85-345F-6D06-B64E-20391C1672CC&auth=3c436cd85e6f7f7a73ca8613b640f4e6e0edaf42-934209ba34ed0e863a26b99cfc784625fd4984d4>

***Response for 3***

We sincerely thank Reviewer 3 for the insightful and constructive comments, which have substantially improved the theoretical clarity and interpretive strength of our manuscript. In response, we have significantly reinforced the theoretical foundation of the study by elaborating on the economic mechanisms that may drive the observed interactions between clean energy and tourism stock indices.

Specifically, we now discuss how ESG-oriented capital flows, policy-induced investment signals, and cost-risk dynamics within tourism operations can jointly contribute to the financial co-movement between these sectors. To further ground our framework, we incorporate the theoretical perspective of Pástor, Stambaugh, and Taylor (2022), who model how ESG preferences influence asset prices and investor behavior. Their insights support the interpretation of clean energy indices as forward-looking sustainability signals that may affect tourism sector valuations, especially in environmentally sensitive or policy-driven markets. (p. 9, L 352).

We have also revised our interpretation of the quantile causality results by drawing on portfolio theory and behavioral finance. In particular, we now explain why significant predictive relationships appear predominantly in the lower and upper quantiles, where investor behavior tends to deviate from rational expectations due to fear, optimism, or capital reallocation under stress or opportunity. We recognize the theoretical value that behavioral finance offers in understanding nonlinear investor responses. However, our objective in this study is not to construct a formal behavioral model, but rather to interpret these patterns at a conceptual level. In particular, we now explain why significant predictive relationships appear predominantly in the lower and upper quantiles, where investor behavior tends to deviate from rational expectations due to fear, optimism, or reallocation under stress or opportunity (p.23-24-25).

Regarding the wavelet coherence analysis, we acknowledge the previous lack of contextual depth and have expanded the discussion with country-specific economic narratives. These include China’s stimulus-led post-COVID recovery, the United States’ decentralized policy response, Europe’s uneven energy transition and regulatory fragmentation, and Australia’s strong domestic tourism rebound supported by energy self-sufficiency and ESG-aligned investment practices (p. 30-35).

We believe these additions closely address the reviewer’s recommendations and help frame the empirical results within a coherent and meaningful economic narrative. We are once again grateful for the reviewer’s valuable input, which has meaningfully strengthened both the theoretical and interpretive dimensions of the paper.

**Reviewer’s concern:**

The reviewer noted the absence of a strong theoretical rationale linking clean energy and tourism sectors. They requested that a clearer economic mechanism be developed to explain why these sectors might be connected and how observed empirical patterns make economic sense.

**Response:**

We fully agree with the reviewer that the theoretical rationale should be more explicit. In the revised introduction and discussion sections, we now anchor our analysis in two main theoretical frameworks: (p.2 and 4; p. 40-42) Portfolio Diversification Theory: Clean energy stocks may act as hedges or substitutes under market stress, influencing tourism sector returns asymmetrically during periods of volatility.

Environmental Kuznets Curve (EKC): As economies develop and transition toward sustainability, sectors like tourism and clean energy may become financially co-integrated due to policy alignment and shared ESG imperatives.

In addition, in the discussion, we relate quantile-specific findings to investor behavior under asymmetric information and crisis response, and interpret wavelet-based co-movements in light of region-specific policies and pandemic recovery trajectories.

We fully agree with the reviewer that a stronger and more explicit theoretical foundation is essential to justify the relationship between clean energy and tourism sectors. In the revised manuscript, particularly in the introduction and discussion sections, we now anchor our analysis in two key theoretical frameworks:

- Portfolio Diversification Theory suggests that clean energy assets may serve as hedging instruments or substitutes during periods of market volatility.

As tourism stocks are particularly sensitive to global shocks, ESG-aligned clean energy investments can attract reallocations of capital during times of stress or uncertainty, resulting in asymmetric co-movements.

- Environmental Kuznets Curve (EKC) logic implies that as economies transition toward sustainability, clean energy and tourism sectors may become increasingly financially integrated. This convergence stems from overlapping policy incentives, shared ESG expectations, and the growing role of green investment strategies in shaping sectoral performance. In addition, we provide contextual interpretations of quantile-specific results by referring to stress-related capital shifts and asymmetries in sectoral exposure across regions. Similarly, wavelet-based temporal patterns are discussed in relation to region-specific policy responses and post-pandemic recovery trajectories. We believe these enhancements offer a more coherent economic rationale for the observed empirical findings and align the theoretical framework with the reviewer’s recommendations

**Response to Reviewer 2**

We sincerely thank Reviewer 2 for their thoughtful and constructive feedback, which has greatly helped improve the quality and clarity of our manuscript. Below we respond point-by-point to each of the comments raised.

***Comment 1:*** Abstract lacks clarity and proper structure

**Reviewer’s concern:** The abstract does not fully follow a logical sequence specifically, it lacks a clearly defined motivation, methodological explanation, and policy relevance. The reviewer recommended organizing it by first stating the research question and motivation, then literature background, methodology, results, and finally policy implications.

**Response:**

We thank the reviewer for the detailed and constructive feedback regarding the structure and clarity of the abstract. We fully agree that a well-organized abstract should follow a logical progression that clearly communicates the motivation, methodological approach, key findings, and policy relevance of the study.
In response to the reviewer’s recommendation, we have thoroughly revised the abstract to reflect a more structured academic format. The revised abstract now opens with a clear statement of the research motivation and question, followed by a concise articulation of the literature gap, emphasizing the limited empirical exploration of financial linkages between clean energy and tourism sectors—particularly acrossregions.
We then provide a succinct overview of the methodological framework, highlighting the use of quantile Granger causality, wavelet coherence, and structural VAR models to uncover asymmetric and time-varying relationships. The main findings are summarized, with emphasis on the heterogeneous patterns identified at extreme quantiles and across regions.

Finally, the abstract concludes with a clear articulation of the study’s practical implications, particularly regarding region-specific investment and sustainability policy strategies.
We believe the revised version more effectively communicates the scope, methods, and significance of our research, and aligns closely with the reviewer’s expectations.

Thank you for this suggestion. We have thoroughly revised the abstract to reflect the structure you recommended. The new version now:

- Opens with a clear research gap and motivation,
- Briefly mentions existing literature,
- Clearly states the methodology (including sample period and data frequency),
- Highlights the key findings in both quantile and time-frequency domains,
- Concludes with practical implications for investors and policymakers.

This new abstract improves clarity and emphasizes the originality and contribution of our work.

***Comment 2:*** Improve clarity and placement of research questions and hypotheses

**Reviewer’s concern:** The research questions were originally placed in Section 2.4, which made the introduction lose focus. The reviewer advised moving these to the introduction and formulating testable hypotheses.

**Response:**

We agree with the reviewer’s concern. The research questions have now been moved to the final paragraph of the Introduction section (p 4-5, L 124-139). In addition, four explicit hypotheses have been formulated and clearly listed. These hypotheses reflect the directional, regional, and asymmetric dynamics we aim to test in the empirical analysis.

***Comment 3:*** Introduction is too general and repetitive

**Reviewer’s concern:** The introduction contains repetitive general statements (e.g., tourism’s contribution to GDP) without regional differentiation or quantification.

**Response:**

We have streamlined the introduction to eliminate generalities and reduce repetition. The revised version now includes region-specific justifications for including Europe, the U.S., Australia, and China, supported by recent statistics and policy references. This restructuring tightens the narrative arc and focuses more directly on the gap our study addresses (p 2,4 L 44-52).

***Comment 4:*** Literature review is not analytical or up to date

**Reviewer’s concern:** The literature section is broad but lacks synthesis or critique. Also, only ~49% of the references are from the last five years.

**Response:**

We have updated the literature review extensively, incorporating multiple new studies published between 2022 and 2024, such as Avcı et. al. (2025), Athari & Kirikkaleli (2024) and Zeng et al. (2024).

Additionally, each subsection now includes comparative evaluations of findings across papers, notes methodological differences, and clarifies how our study offers a novel contribution. A revised subsection has been added to explicitly highlight the literature gap, including what remains unexplored in inter-sectoral financial relationships (p 5-8).

***Comment 5:*** Discussion repeats results; needs theoretical and comparative interpretation

**Reviewer’s concern:** The discussion section mainly repeats findings without interpreting them through theoretical frameworks (e.g., portfolio theory or EKC), economic mechanisms, or comparisons with the literatüre.

**Response ( p 36-42):**

- This section has been extensively revised. The updated discussion now:
- Interprets quantile results via portfolio diversification behavior and asymmetric investor reactions,
- Explains regional variations through policy structure, ESG investment climate, and market resilience,
- Links wavelet findings to specific events (e.g., post-COVID-19 divergence, energy price shocks),
- And compares our findings to earlier empirical studies (e.g., Wang et al., 2025; Zeng et al., 2024).

***Comment 6:*** Recommendations are vague; no stakeholder differentiation

**Reviewer’s concern:** The recommendations were broad and non-actionable. The reviewer asked for more concrete guidance, separating suggestions for governments, investors, and tourism firms.

**Response:**

We have completely revised the “Conclusion and Recommendations” section. It now includes clear, actionable suggestions tailored to three stakeholder groups:

- Governments: Integration of clean energy incentives into tourism recovery plans,
- Investors: Portfolio strategies using clean energy as a hedge in tourism-exposed regions,
- Tourism firms: ESG compliance and strategic alignment with clean energy trends.
- Each recommendation is tied directly to the empirical evidence.

***Comment 7:*** Limitations are not discussed

**Reviewer’s concern:** The study does not mention its regional and methodological limitations, particularly the exclusion of emerging economies like Brazil or India.

**Response:**

A paragraph on limitations has been added to the Conclusion. We now acknowledge that the analysis is confined to four major economies and excludes important emerging markets. We also note that future research should expand the regional scope and incorporate firm-level and ESG-specific variables. Additionally, we mention that structural identification methods were not pursued in this study but are a useful extension.

***Comment 8:*** *Minor English issues*

**Reviewer’s concern:** Although the language is mostly clear, the reviewer recommended minor polishing for clarity, especially in the methodology and discussion.

**Response:**

We have carefully revised the entire manuscript for clarity and consistency. Additional editing was conducted in the methodology, discussion, and abstract sections to enhance conciseness and readability. We are open to further professional editing upon acceptance if required by the journal.

**Note:** We have enabled the ‘Track Changes’ feature in the manuscript file and highlighted all newly added text in yellow.

Furthermore, the manuscript title and keywords have been updated in accordance with the reviewers’ suggestions.
